# Monoamine oxidase a gene polymorphism associated with hostility in male population of 45-64 in Russia/Siberia

**DOI:** 10.1192/j.eurpsy.2021.1173

**Published:** 2021-08-13

**Authors:** V. Gafarov, E. Gromova, D. Panov, I. Gagulin, A. Gafarova, V. Maximov

**Affiliations:** Collaborative Laboratory Of Cardiovascular Diseases Epidemiology, Institute of Internal and Preventive Medicine - branch of Institute of Cytology and Genetics SB RAS, Novosibirsk, Russian Federation

**Keywords:** population, men, MAOA gene, hostility

## Abstract

**Introduction:**

The presence of low-activity alleles of the MAOA gene increases the risk of hostility.

**Objectives:**

To study the association of hostility with high and low-active variants of the MAOA gene in an open population of men 45-64 years.

**Methods:**

Under the WHO International Program MONICA-psychosocial and HAPIEE a representative sample of men aged 45–64 years (n = 781 men, average age was 56.48 ± 0.2 years) examined in 2003-2005. All respondents independently completed a questionnaire on hostility. From the surveyed sample using the random number method 156 men were selected who were genotyped for MAOA-uVNTR polymorphism.

**Results:**

It was found the level of hostility in the population of men was 60.3%. In persons with low-active alleles of the MAOA-L gene (allele 2 and 3) a high level of hostility was more common - 50.9%. The results of building a logistic regression model showed that the presence of low-active alleles (2; 3) of the MAOA gene increases the likelihood of hostility OR = 2,103 (95% CI 1,137-3,889, p = 0.018).

**Conclusions:**

Our findings allow us to conclude that the low-active allele of the MAOA-L gene is associated with hostility.

